# A community-partnered approach for diversity in COVID-19 vaccine clinical trials

**DOI:** 10.1017/cts.2022.471

**Published:** 2022-10-06

**Authors:** Yelba Castellon-Lopez, Raphael Landovitz, Ejiro Ntekume, Courtney Porter, Rachelle Bross, Robin Hilder, Aziza Lucas-Wright, Eric S. Daar, Pedro Chavez, Christopher Blades, Savanna Carson, D’Ann Morris, Stefanie Vassar, Alejandra Casillas, Arleen Brown

**Affiliations:** 1 Department of Family Medicine, UCLA David Geffen School of Medicine, University of California, Los Angeles, CA, USA; 2 Division of Infectious Disease, David Geffen School of Medicine, University of California, Los Angeles, CA, USA; 3 Division of General Internal Medicine and Health Services Research, Department of Medicine, David Geffen School of Medicine, University of California, Los Angeles, CA, USA; 4 The Lundquist Institute at Harbor-UCLA Medical Center, Torrance, CA, USA; 5 Charles R Drew University of Medicine and Science, Los Angeles, CA, USA; 6 UCLA Vine Street Clinic, Los Angeles, CA, USA

**Keywords:** COVID-19, community engagement, research subject recruitment, vaccine trials, health equity

## Abstract

**Introduction:**

Communities of color have faced disproportionate morbidity and mortality from COVID-19, coupled with historical underrepresentation in US clinical trials, creating challenges for equitable participation in developing and testing a safe and effective COVID-19 vaccine.

**Methods:**

To increase diversity, including racial and ethnic representation, in local Los Angeles County NIH-sponsored Phase 3 SARS-CoV-2 vaccine clinical trials, we used deliberative community engagement approaches to form a Community Consultant Panel (CCP) that partnered with trial research teams. Thirteen members were recruited, including expertise from essential workers, community-based and faith-based organizations, or leaders from racial and ethnic minority communities.

**Results:**

Working closely with local investigators for the vaccine studies, the CCP provided critical insight on best practices for community trust building, clinical trial participation, and reliable information dissemination regarding COVID-19 vaccines. Modifying recruitment, outreach, and trial protocols led to majority–minority participants (55%–78%) in each of the three vaccine clinical trials. CCP’s input led to cultural tailoring of recruitment materials, changes in recruitment messaging, and supportive services to improve trial accessibility and acceptability (transportation, protocols for cultural competency, and support linkages to care in case of an adverse event). Barriers to clinical trial participation unable to be resolved included childcare, requests for after-hours appointment availability, and mobile locations for trial visits.

**Conclusion:**

Using deliberative community engagement can provide critical and timely insight into the community-centered barriers to COVID-19 vaccine trial participation, including addressing social determinants of health, trust, clinical trial literacy, structural barriers, and identifying trusted messenger and reliable sources of information.

## Introduction

The use of vaccines to prevent Coronavirus disease 2019 (COVID-19) is critical to the reduction of disproportionate pandemic-related morbidity and mortality in racial and ethnic minority communities that have seen declines in life expectancy due to COVID-19 [1–4]. Early in the pandemic, there were low racial and ethnic minority participation rates in phase I and II clinical trials [5]. Historical underrepresentation of minorities in clinical trials [6,7], including vaccine trials [8,9], presented a critical challenge to the successful development, testing, and use of a safe and efficacious vaccine by those who need it most. Underrepresentation of participants from diverse racial and ethnic backgrounds may have implications for the generalizability of clinical trial results, given the efficacy and safety of medical treatments may differ by race or ethnicity [10]. Enhanced representation of diverse groups in vaccine trials may also enhance subsequent vaccine uptake [5], increase equitable access to other timely or novel treatments, and contribute to our understanding of health disparities. Thus, early in the inception of COVID-19 vaccine trials, the US National Institutes of Health (NIH) identified increasing accessibility to underrepresented populations as a priority and vital to providing equitable protection from COVID-19.

To address enrollment gaps in COVID-19 clinical trials, we collaborated with NIH-sponsored Phase 3 COVID-19 vaccine trial teams in Los Angeles County to convene a Community Consultant Panel (CCP), an advisory group designed to provide community feedback and recommendations to improve the recruitment and retention of minority participants. The goals of the CCP were to help increase participant diversity and representation – by race/ethnicity, essential worker occupation, and geography by recruiting CCP members from underrepresented racial and ethnic groups from communities with high rates of infection, morbidity, and mortality – in local COVID-19 vaccine clinical trials and provide access to accurate information about clinical trials to communities disproportionately affected by COVID-19. Assuring diversity among trial participants was particularly relevant as California has the highest number of COVID-19 cases nationwide, with 32% of cases in Los Angeles County, one of the country’s most populated and racially diverse counties [11]. We describe our approach to forming the CCP, how recruitment strategies were modified, and enrollment outcomes across three local vaccine trials. We summarize actionable strategies recommended by the CCP to improve the engagement of minority populations in COVID-19 vaccine trials within our academic institutions.

## Materials and Methods

The CCP was rapidly formed by the UCLA Clinical and Translational Research Institute’s (CSTI) Community Engagement & Research Program (CERP) to support three COVID-19 clinical trial research teams across Los Angeles County with clinical trial recruitment and retention (UCLA CARE Center, Harbor UCLA/Lundquist Institute, and UCLA Vine Street Clinic) enrolling into two NIH-funded COVID-19 clinical trials (AstraZeneca and Moderna). The CCP had several roles: to consult with academic clinical trial researchers, health professionals, and other Los Angeles-based community leaders, to identify barriers and facilitators to COVID-19 vaccine clinical trial participation across diverse communities in Los Angeles County, and to provide recommendations for enhancing clinical trial participation in diverse communities.

### Deliberative Community Engagement

We used a Deliberative Community Engagement (DCE) approach to understand and enhance clinical trial recruitment and implementation and better understand and address the pervasive lack of diverse representation in clinical trials. DCE has been used to examine and obtain community input on a variety of complex health and social issues [12–15]. The process allows participants to consider relevant information from multiple points of view and involves: recruiting a sample of relevant stakeholders (regarded as experts in how the topic at hand concerns or affects the population at risk) to serve as deliberates; engage in educational activities to ensure stakeholders have a working knowledge of the technical issues at hand, as well as clinical, social, and other trade-offs; facilitate discussion so participants can clarify their values and understand others’ perspectives; and develop and discuss specific recommendations [12].

### CCP Member Recruitment

Representatives from communities with a high risk of COVID-19 due to race/ethnicity and/or age, occupation (e.g., essential service industries), or geographic region served were identified by members of the CERP academic-community collaborative based on occupations, regions, poverty level, and ages with higher than average COVID-19 cases reported by Los Angeles County. A list of recommended candidates was circulated to the CERP community partners and academic faculty, who then ranked individuals to ensure representation from all potential professional sectors and communities (race/ethnicity, occupation type or organization, and geographic area) as well as noting any previous experience working with the individual such as previous CBPR projects, CTSI community partners, and personal and professional networks. The clinical trial team tabulated, reviewed, and discussed ratings to select 13 candidates. Invitations were sent to panelists defining the role of the consultant, participation expectations, and compensation.

### CCP Structure and Curriculum

Because of the immediate need to implement trial protocols and design equitable engagement plans, CCP met weekly over zoom with UCLA researchers for eight weeks (90 min per meeting) in Summer 2020 to identify barriers to trial participation. Members were compensated for their time commensurate with the expected time commitment, both during and outside the scheduled meetings, and were offered a tablet with free internet access if needed for CCP participation. Before the first meeting, each participant received a clinical trial briefing booklet with information on COVID-19, clinical trial stages and processes, the importance of diverse participation in research, and the protection of human subjects. These and other materials were also made available to participants on a website, which was regularly updated with evolving information.

Weekly CCP meetings aimed to promote bidirectional exchange on trial processes, COVID-19, vaccines, and other topics determined by the CCP. The academic team (principal site investigators, clinical trial staff, and CERP staff) developed and shared brief educational presentations about COVID, vaccine development, regulatory approval, emergency authorization processes, and vaccine risk, benefits, and safety. Discussions were framed around community concerns related to trial participation, strategies for clear and culturally appropriate recruitment messaging, and trial participation barriers of high-risk groups. Discussions were driven by new information about the virus, vaccines, trials, and other topics determined by the CCP. Supplemental Table 1 provides a brief outline of the educational objectives of each session and the discussion topics covered during the CCP meeting. For some of the sessions, representatives from a marketing and communications firm hired by two of the local clinical trials participated and had an opportunity to interact directly with community members on language and design of outreach materials, approach, and potential outlets for messaging. The meetings were recorded, and an academic team member took notes during each session.

Clinical trial investigators reviewed the CCP feedback, discussed the feasibility of suggestions with the CCP, and identified strategies to incorporate proposed recommendations when possible. For these analyses, we summarize the recommendations from the CCP and whether and how the recommendations were acted upon.

After the last session, CCP participants were invited to complete a survey that included demographic characteristics, previous research or consulting experience, perceived experience as a community consultant, the perceived value of the community-academic team [16], and how they used and disseminated information gained through the CCP. A five-point Likert scale was used to gauge the success of each metric.

### Cross-Collaboration across COVID-19 Vaccine Clinical Trial Leads in Los Angeles County

In addition to the CCP meetings, the three participating interdisciplinary vaccine trial teams met in weekly 60-min Zoom sessions to share experiences. Investigators used these meetings to share approaches to recruitment and engagement, help answer questions raised during the CCP meetings, and incorporate feedback from the CCP with the vaccine trial research teams.

## Results

The final community panel included 13 participants (Table 1). Participants identified themselves as Black (31%), Latino (39%), Asian (15%), or White (15%). Participants self-identified as representing the following community sectors and special populations: community health workers (54%), essential workers (46%), health care professionals (31%), low-income (69%), individuals with chronic conditions (39%), and LGBTQ (23%). Most participants had experience serving on a community advisory board (69%).

**Table 1.** Community Consultant Panel (CCP) demographics and experiences (N = 13)

Demographics	N (%)
Age	
20–45 years	3 (23.1)
46–64 years	8 (61.5)
Over 65 years	2 (15.4)
Education	
Some college/Associates Degree	2 (15.4)
Bachelor’s Degree	4 (30.8)
Post graduate (Master’s Degree, PhD, etc)	7 (53.8)
Female	8 (61.5)
Race/Ethnicity (check all that apply)	
Latino/Latina/Hispanic	5 (38.5)
Black/African American	4 (30.8)
Asian	2 (15.4)
White	2 (15.4)
Community sectors (check all that apply)	
Community Health Workers/Promotoras	7 (53.8)
Essential workers	6 (46.2)
Healthcare Professionals	4 (30.7)
Other: patients (1), older adults (1), labor trafficking survivor (1), non-profit organizations (2)	5 (38.5)
Population you feel you represent (check all that apply)^a^	
Black/African American	6 (46.2)
Hispanic/Latino	5 (38.5)
Asian American or Pacific Islander	2 (15.4)
Low-income	9 (69.2)
Chronic Disease	5 (38.5)
Lesbian, Gay, Transgender, Bisexual, Queer/Questioning	3 (23.1)
Other: Immigrants (1), Faith-based (1), Older adults (1)	3 (23.1)
Prior research experience (check all that apply)	
Previously served on a Community Advisory Board^b^	9 (69.2)
Worked on a research study with an academic institution	8 (61.5)
Satisfaction with CCP participation^c^	
Felt welcome as a member of the CCP	12 (92.3)
Felt comments were valued by the academic team	12 (92.3)
Gained new information about clinical trials, COVID-19, or COVID-19 vaccines	13 (100.0)
Applied new information outside of the CCP	13 (100.0)

a Provided options were: Black/AA, LatinX/Latino, White/Caucasian, Asian/Pacific Islander, Native American, LGBTQ, Low income, Chronic Disease, Other (please specify).

b May include community advisory boards for academic or non-academic institutions.

c Response of “Very Satisfied” or “Extremely Satisfied” on a Likert scale.

All the participants reported gaining new information about clinical trials, COVID-19, and/or the COVID-19 vaccine due to CCP participation. All thirteen participants reported applying the new information they learned in the community, including sharing the information with family (85%), friends (77%), co-workers (92%), and others (8%). The vast majority, 92%, reported that they felt welcomed as a member of the CCP, and 92% felt that the academic team valued their comments (Table 1).

### Actionable Strategies Incorporated into Local COVID-19 Vaccine Trials

Clinical trial investigators could incorporate several proposed recommendations (Table 2). The recommendations addressed three domains. First, the participants recommended increasing trust and transparency in the research process by clarifying vaccine science or trial processes, utilization of trusted messages and messengers, and highlighting trial participation benefits in addition to risks. The second group of suggestions related to the importance of inclusive community-engaged approaches to promote more effective outreach and recruitment, developing culturally tailored approaches for engagement, and recruitment communications that promote inclusion, including engagement strategies for responding to questions, concerns, misinformation, and disinformation prevalent in the community. Third, the CCP strongly endorsed enhancing trial accessibility and acceptability related to the social determinants of health, including improving local practices that promote a welcoming environment and address the needs of low-income individuals with competing financial and social demands and study protocols. They specifically recommended strategies to reduce barriers to participation, such as providing transportation services, language translation services for visits and all study documents, offering child care services, and addressing concerns about access to medical visits for vaccine-related side effects for those who lack health care coverage. CCP members specifically endorsed the importance of creating a welcoming study environment through intentional “customer service” efforts to improve trust, acceptability, and retention of underrepresented populations in research. The panel recommended that participants receive access to food and refreshments, accommodations for additional instruction, and a clear plan for visits and follow-up to address health literacy, language translation services, and recognition for their participation and time. Lastly, participants recommended clinical trial sites be located directly in the community to improve accessibility and reduce social burden.

**Table 2.** Community Consultant Panel (CCP) recommendations for improved participation in clinical trials and modifications made by investigators

Domains of discussions from Community Panel	Specific trial modifications developed in collaboration with the CCP, including implemented strategies and recommendations that could not be implemented (denoted with ^a^)
*Increase trust in the research process*	
Enhance transparency	• Indicate source(s) of trial funding (private vs. public/NIH) and of decision-making power for discussed modifications, i.e. local vs. national vs. pharmaceutical companies • Transparent trial-relevant information readily available to public in lay language (COVID-19, vaccine trial processes, vaccine types and status) • Trial participation expectations and procedures (time in participation, compensation, risks, and benefits)
Need for trusted messages with accurate information	• Identify trusted community venues, media, and leaders for outreach about COVID-19, clinical trials, and vaccines • Support messengers with clear information and sources about COVID-19, vaccine trial processes, and vaccine types
*Community engagement and recruitment strategies*	
Engagement and outreach	• Multiethnic media and venues for trusted communication • Approaches (face-to-face, virtual, written, flyers, social media) • Community-identified venues and leaders to share clinical trial opportunities and provide informational sessions • Appropriate language and messaging based on race/ethnicity, literacy-level, translation, or cultural context for outreach
Suggestions for local clinical trial recruitment website	• Customize website for usability, accessibility, readability, inclusive language, benefits of inclusion, and common community questions • Increase diversity and representation of minority groups in website images • Improved FAQ questions section addressing participant expectations in clinical trials
Suggestions for outreach materials (flyer) for clinical trials	• Create a Spanish version of the trial outreach flyer, an additional Spanish-language recruitment website, screening, and consent materials • Tailor language on materials (increase font for readability, remove scientific jargon, and use appropriate reading level) • Use inclusive wording (i.e., instead of, “help us find a COVID-19 vaccine,” use, “Let’s find a COVID-19 vaccine together” • Remove language on “documentation of negative COVID19 test” to reduce the opportunity of potential confusion for undocumented participants • Add complete contact information for questions and inquiries (phone, website, email, and language translation services) rather than only the website to sign-up • Use images reflecting a diverse population
Address emergent issues and misinformation around COVID-19 vaccine trials	• Participate in community discussions to update community members and to help address mis- and dis-information identified by CCP and their networks
*Enhance trial accessibility and acceptiblity*	
Provide transportation for participants	• Provide transportation for routine visits and COVID-19 positive “sick day visits”
Provide a welcoming environment and “customer service” at clinical trials sites	• Provide descriptions of what to expect when arriving at the sites, language concordant greetings and introductions, familiarization or tour of location (where to find restrooms, waiting areas, etc.), and genuine appreciation of trial participation • Provision of snack bags/water bottles upon entrance or exit • Obtain patient preferences (pronouns, name, language) • Adjust/reduce waiting times for participants • Include diverse staff members to great participants and language concordant staff (and for phone calls) • Include child/elder care during visits^a^ • Provide other health services at the clinical trial sites^a^ • Clear explanations and communication of eligibility and ineligibility
Locate trial sites in minority communities	• Recommended additional trial sites in neighborhoods with high COVID-19 risk and outcomes^a^
Access to health care for uninsured participants if adverse reactions/COVID related illnesses	• Emphasize that participants receive health coverage for study-related injuries and adverse reactions through the National Vaccine Injury Compensation Program • Health coverage for COVID-19 clinical care is not included^a^ • Referral protocols for uninsured or underinsured persons to obtain needed COVID-19 or other clinical care

a Recommendations the Los Angeles COVID-19 trial teams were unable to implement (see narrative for additional detail).

Discussions allowed for the iterative development of locally tailored strategies to modify engagement practices related to outreach, recruitment, and retention. During these discussions, the panelists identified individuals, agencies, organizations, media outlets, and community members they viewed as trusted messengers, provided introductions to key stakeholders in the community, and helped craft messages for trial information and dissemination. To enhance transparency, the team shared information on NIH and industry funding for the trials in Los Angeles County and links to websites with that information. Structured discussions were held on trial protocols for each vaccine (“clinical trial basics”), participant expectations related to the timing of procedures, vaccine types, risks such as side effects and the potential for lack of efficacy, as well as the potential benefits of participation, including compensation, early access to promising vaccines, and medical screenings.

The panelists and the study teams developed, tested, and customized messaging for different communities in Los Angeles County to improve recruitment. Investigators and panelists collaborated on 112 community discussions for organizations represented by the CCP members, including churches, Filipino social clubs, LGBTQ podcasts, and parent organizations, reaching over 10,000 individuals. Due to the pandemic, many of these events occurred virtually.

Tailoring of the recruitment messaging incorporated readability, inclusive language, diversity in images of participants represented, and updating frequently asked questions in response to trial and vaccine development progress, changes in the pandemic, and CDC and local public health guidance. For example, initial drafts of clinical trial recruitment materials listed eligibility criteria indicating the need for “documentation” of a negative COVID-19 test. The CCP noted the word “documentation” was a potential trigger for deterring undocumented persons or families with mixed documentation status from participation due to the burden of proof and misconceptions participation in public benefits (access to free COVID-19 test) can interfere with future immigration eligibility, also known as fear of public charge [17,18]. Ensuring undocumented populations felt safe in clinical trial enrollment was particularly important to the CCP, considering it was unknown at that time if COVID-19 vaccines would only be covered by health insurance.

To improve accessibility and acceptability for trial participation, participants suggested ways to reduce participation barriers related to social determinants of health and suggested improving trial retention through a genuine focus on the participant. Social barriers were mitigated by providing transportation for participants (both for routine visits and “sick day visits,” e.g., visits to the clinical trial site to address symptoms that might represent infection or side effects related to the vaccine), Spanish translation services, and referrals to resources for those who were uninsured or underinsured. Although most of the participating clinical trial sites had a strong record of collaboration with diverse communities, the extensive “customer service” recommendations from the CCP were important reminders of the need to build rapport with participants to enhance retention. Recommendations included a welcoming environment for participants, many of whom had never participated in a clinical trial yet were now doing so in the context of COVID-19 distancing requirements and other restrictions, increased personal and community stressors, and competing clinical and social demands brought about by the pandemic. Specific recommendations included thanking participants for their time, providing clear directions before their appointment, a tour and introductions, asking about gender pronouns, and supplying water, snacks, or a “goodie bag.”

The local clinical trials could not address some CCP recommendations. For example, participants strongly endorsed the need for more clinical trial locations in minority communities through mobile trial sites and partnerships with minority services institutions. However, most vaccine trial sites were identified based on prior NIH accreditation, and available mobile vans could not process the clinical trial samples. Other suggestions, such as a need for after-hours availability, including weekends and weeknights, and on-site child care, were not feasible due to union, staffing, or resource limitations. Finally, some panelists’ inadequate staff diversity at some sites and lack of fluency in languages other than English and Spanish were major concerns, particularly for representatives of Asian and Pacific Islander communities. Panelists and clinical research teams endorsed the need for lay health workers from these communities who could facilitate participation in the participant’s preferred language, study materials (including consent forms and informational materials), and staff and/or translators who could address these participants’ needs in real time.

### Recruitment of Diverse Communities in the Local COVID-19 Vaccine Trial

**Fig. 1.**
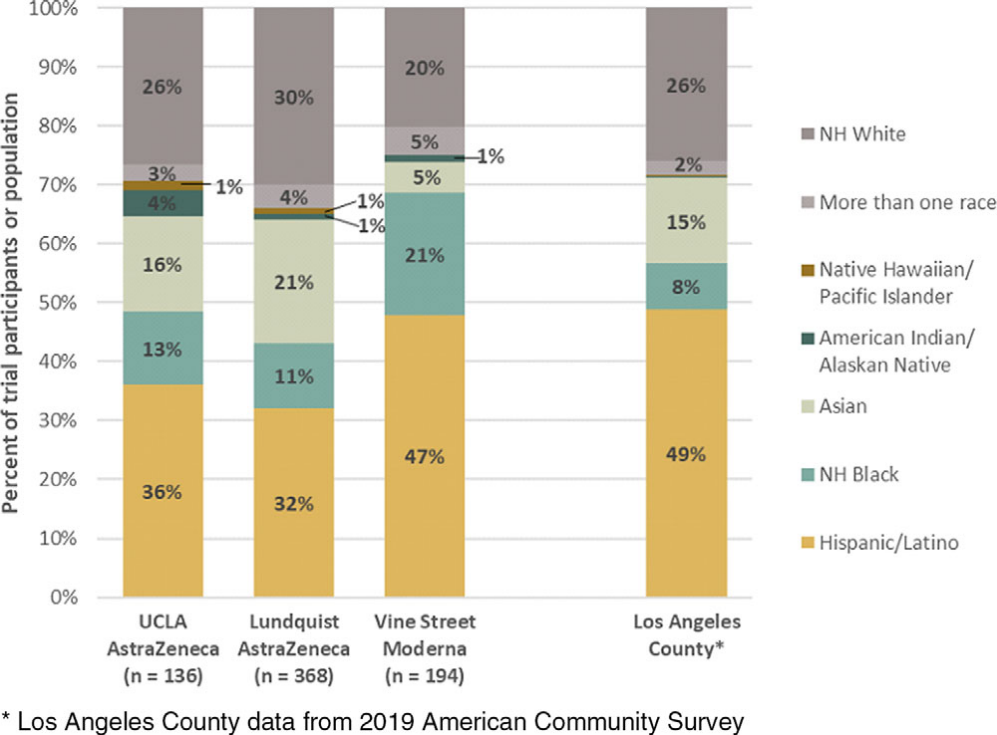
Racial and ethnic composition of participants enrolled in partnered clinical trials compared to Los Angeles County population. *Los Angeles County data from 2019 American Community Survey.

Deploying several of the CCP’s recommendations for trial engagement, recruitment, messaging, accessibility, and acceptability, our three local trials reported more than 50% underrepresented minority participation, with the following ranges: 32%–47% Latino, 20%–31% White, 11%–21% Black/African American, 5%–21% Asian American, 2% Native Hawaiian or other Pacific Islander, 1%–4% American Indian/Alaska Native, and 0.5%–5% Other/Multiracial. The racial and ethnic breakdown of local COVID-19 vaccine clinical trial enrollment closely mirrored the racial and ethnic composition of Los Angeles County (Fig. 1) and showed larger proportions of racial and ethnic minorities than the aggregate Moderna and AstraZeneca trial enrollments [19,20].

## Discussion

The disparities in COVID-19 and mortality rates in communities of color represent longstanding systemic health inequities [21,22]. Underrepresentation of minorities in COVID-19 clinical trials may result in limited generalizability of outcomes and decreased vaccine confidence and uptake among communities most impacted by the COVID-19 pandemic. We describe a community-engaged approach to developing community-centered recommendations to improve racial and ethnic diversity in COVID-19 vaccine clinical trials. We found that in addition to leveraging dedicated resources to help vulnerable communities overcome barriers related to the social determinants of health, engagement is critical for reaching diverse participant pools, building trust and transparency, and reducing obstacles to participation. Others have also advocated for the implementation of strategies leveraging community-partnered research [5], such as partnering with Black church leaders and other trusted community leaders [23], and acknowledging the role of racism and history of systematic abuse and mistreatment both in health care and medical research for racial and ethnic minorities in the USA [24].

The DCE approach to COVID-19 vaccine trial recruitment presented an opportunity to understand and reduce community participation barriers, address informational needs, and improve acceptability. Some suggestions put forth by the CCP were adopted through modifications to each clinical trial team’s recruitment approach. Although we could not incorporate all CCP recommendations, the diverse panelists, investigators, and staff allowed for robust discussions of the policies and practices needed to effect long-term, fundamental change in the planning for and implementation of clinical trials in nontraditional settings to engage more diverse participant populations.

Our project had some limitations. This process relied on longstanding community ties and may be difficult to replicate. The rapid implementation timeline also required significant funding to support the DCE approach, staff trained in community engagement, and adequate compensation for CCP participants, given the demands on their time and the need for a quick turnaround for feedback. This study took place in a racially and ethnically diverse urban setting with access to several recruitment sites, so it may not generalize to other locations. Finally, many CCP members had prior experience with research and were highly educated. The perspectives of the members of the CCP may not represent the community at large or those with less favorable views of research; however, we intentionally recruited community leaders with experience working with high-risk communities and racially and ethnically diverse community members to inform our approach. While recruiting diverse participants in clinical trials is essential, future research should focus on retention strategies for participants from underrepresented groups in underresourced communities.

Our results have important policy implications. High-risk communities should be involved in clinical trial planning to address the profound health disparities during the COVID-19 pandemic. An established community partner network, organizational infrastructure, and leadership that supports this process allowed us to leverage trusted relationships from a vast network of community stakeholders. The panelists were able to effectively collaborate with clinical trial leadership and staff to provide insight and practical advice on community concerns, share updated and valuable information for their communities, and enhance researchers’ awareness of unique barriers and facilitators to participation in COVID-19 vaccine clinical trials from the perspective of diverse local communities. To promote the generalizability of clinical trial outcomes and address the needs of populations at the highest risk for health inequities, policies are needed to enhance representation in the biomedical workforce, promote collaborations with trusted community members and organizations, and develop and monitor metrics for diversity in clinical trials beyond race/ethnicity, age, and gender (i.e., socioeconomic status, insurance status, sexual identity and orientation, languages spoken, language preferences). Such policies will build confidence, engage community stakeholders early in the clinical trial process, and overcome social disparities that contribute to health inequities. Ensuring ample funding for community investment and capacity building to create mutually beneficial and reciprocal relationships between researchers and communities is essential to improve the representation of diverse communities in clinical trials.

## Supplementary material

For supplementary material accompanying this paper visit https://doi.org/10.1017/cts.2022.471. click here to view supplementary material

## References

[1] Killerby ME , Link-Gelles R , Haight SC , et al. Characteristics associated with hospitalization among patients with COVID-19 - Metropolitan Atlanta, Georgia, March–April 2020. MMWR. Morbidity and Mortality Weekly Report 2020; 69(25): 790–794. 32584797 10.15585/mmwr.mm6925e1 PMC7316317

[2] Stokes EK , Zambrano LD , Anderson KN , et al. Coronavirus disease 2019 case surveillance - United States, January 22–May 30, 2020. MMWR. Morbidity and Mortality Weekly Report 2020; 69(24): 759–765. 32555134 10.15585/mmwr.mm6924e2 PMC7302472

[3] Dorn AV , Cooney RE , Sabin ML. COVID-19 exacerbating inequalities in the US. Lancet 2020; 395(10232): 1243–1244. 32305087 10.1016/S0140-6736(20)30893-X PMC7162639

[4] Andrasik MP , Broder GB , Wallace SE , et al. Increasing Black, Indigenous and People of Color participation in clinical trials through community engagement and recruitment goal establishment. PLoS One 2021; 16(10): e0258858. 34665829 10.1371/journal.pone.0258858 PMC8525736

[5] Flores LE , Frontera WR , Andrasik MP , et al. Assessment of the inclusion of racial/ethnic minority, female, and older individuals in vaccine clinical trials. JAMA Network Open 2021; 4(2): e2037640. 33606033 10.1001/jamanetworkopen.2020.37640 PMC7896193

[6] Heiat A , Gross CP , Krumholz HM. Representation of the elderly, women, and minorities in heart failure clinical trials. Archives of Internal Medicine 2002; 162(15): 1682–1688. 12153370 10.1001/archinte.162.15.1682

[7] Hussain-Gambles M , Atkin K , Leese B. Why ethnic minority groups are under-represented in clinical trials: a review of the literature. Health and Social Care in the Community 2004; 12(5): 382–388. 15373816 10.1111/j.1365-2524.2004.00507.x

[8] Djomand G , Katzman J , di Tommaso D , et al. Enrollment of racial/ethnic minorities in NIAID-funded networks of HIV vaccine trials in the United States, 1988 to 2002. Public Health Reports 2005; 120(5): 543–548. 16224987 10.1177/003335490512000509 PMC1497755

[9] Sobieszczyk ME , Xu G , Goodman K , Lucy D , Koblin BA. Engaging members of African American and Latino communities in preventive HIV vaccine trials. Journal of Acquired Immune Deficiency Syndromes 2009; 51(2): 194–201. 19504752 10.1097/qai.0b013e3181990605 PMC4465439

[10] Ramamoorthy A , Pacanowski MA , Bull J , Zhang L. Racial/ethnic differences in drug disposition and response: review of recently approved drugs. Clinical Pharmacology & Therapeutics 2015; 97(3): 263–273. 25669658 10.1002/cpt.61

[11] Health CDoP. *Tracking COVID-19 in CA*. State of California, 2020. (https://covid19.ca.gov/).

[12] Dry SM , Garrett SB , Koenig BA , et al. Community recommendations on biobank governance: results from a deliberative community engagement in California. PLoS One 2017; 12(2): e0172582. 28235046 10.1371/journal.pone.0172582 PMC5325297

[13] Burgess MM. From ‘trust us’ to participatory governance: deliberative publics and science policy. Public Understanding of Science 2014; 23(1): 48–52. 24434712 10.1177/0963662512472160

[14] Garrett SB , Koenig BA , Brown A , et al. EngageUC: developing an efficient and ethical approach to biobanking research at the University of California. Clinical and Translational Science 2015; 8(4): 362–366. 25581047 10.1111/cts.12259 PMC4499012

[15] Abelson J , Blacksher E , Li K , Boesveld S , S. G. Public deliberation in health policy and bioethics: mapping an emerging, interdisciplinary field. Journal of Public Deliberation 2013; 9(1).

[16] Matthews AK , Anderson EE , Willis M , Castillo A , Choure W. A Community Engagement Advisory Board as a strategy to improve research engagement and build institutional capacity for community-engaged research. Journal of Clinical and Translational Science 2018; 2(2): 66–72. 31660220 10.1017/cts.2018.14 PMC6799353

[17] Katz MH , Chokshi DA. The “public charge” proposal and public health: implications for patients and clinicians. JAMA 2018; 320(20): 2075–2076. 30285073 10.1001/jama.2018.16391

[18] Perreira KM , Yoshikawa H , Oberlander J. A new threat to immigrants’ health - the public-charge rule. New England Journal of Medicine 2018; 379(10): 901–903. 30067441 10.1056/NEJMp1808020

[19] Falsey AR , Sobieszczyk ME , Hirsch I , et al. Phase 3 safety and efficacy of AZD1222 (ChAdOx1 nCoV-19) Covid-19 vaccine. New England Journal of Medicine 2021; 385(25): 2348–2360. 34587382 10.1056/NEJMoa2105290 PMC8522798

[20] Baden LR , El Sahly HM , Essink B. Efficacy and safety of the mRNA-1273 SARS-CoV-2 vaccine. New England Journal of Medicine 2021; 384: 403–416. 33378609 10.1056/NEJMoa2035389 PMC7787219

[21] Thompson HS , Manning M , Mitchell J , et al. Factors associated with racial/ethnic group-based medical mistrust and perspectives on COVID-19 vaccine trial participation and vaccine uptake in the US. JAMA Network Open 2021; 4(5): e2111629. 34042990 10.1001/jamanetworkopen.2021.11629 PMC8160590

[22] Carrion D , Colicino E , Pedretti NF , et al. Assessing capacity to social distance and neighborhood-level health disparities during the COVID-19 pandemic. medRxiv 2020. DOI 10.1101/2020.06.02.20120790.

[23] Jaklevic MC. Researchers strive to recruit hard-hit minorities into COVID-19 vaccine trials. JAMA 2020; 324(9): 826–828. 32789501 10.1001/jama.2020.11244

[24] George S , Duran N , Norris K. A systematic review of barriers and facilitators to minority research participation among African Americans, Latinos, Asian Americans, and Pacific Islanders. American Journal of Public Health 2014; 104(2): e16–e31. 10.2105/AJPH.2013.301706 PMC3935672 24328648

