# Supplementary material for: A community-partnered approach for diversity in COVID-19 vaccine clinical trials
Source: J Clin Transl Sci. 2022 Oct 6;7(1):e23. doi: 10.1017/cts.2022.471 (PMC9874035; doi:10.1017/cts.2022.471)
Supplement: Supplementary file 1 [file S205986612200471Xsup001.docx]

**Supplement 1: CCP Session Learning Objectives and Discussion Topics**

| **Session** | **CCP Session Agenda Topics** |
| --- | --- |
| CCP Member Briefing | **Introductory packet contents (emailed and mailed) two weeks before the first session:**   - Welcome packet (letter of appreciation, role of CCP member, compensation details, meeting times and expectations) - Clinical trial briefing booklet (COVID-19 facts, vaccine development basics, importance of diverse participation in clinical trials, protection of clinical trial participants, current COVID-19 vaccine development updates including vaccine types, pharmaceutical companies, and stages) - CCP website (including all of the above informational resources and news) |
| Week 1 | **Presentation Topics:**   - Introductions of community members, clinical trial investigator, and community engagement team - Roles and expectations - Importance of community-engaged research - Overview of COVID-19 vaccine clinical trial   **Discussion Topics:**   - COVID-19 Q&A - COVID-19 clinical trial concerns and questions |
| Week 2 | **Presentation Topics:**   - Enrollment process - Informed consent - National stakeholders and sponsors who are financially and administratively supporting the clinical trial   **Discussion Topic:**   - Q&A on clinical trial and information from the last session |
| Week 3 | **Discussion Topics:**   - Community trust – specific to the COVID-19 vaccine clinical trials - Review of NIH COVID-19 Prevention Network (CoVPN) outreach and recruitment materials and recommendations |
| Week 4 | **Discussion Topics:**   - Risks and benefits of vaccine clinical trial participation - Continued discussion of trust in the community |
| Week 5 | **Presentation Topics:**   - Review of clinical trial recruitment materials and media strategy   **Discussion Topics:**   - Continued conversation of risks and benefits of trial participation - Languages offered by clinical trial staff and potential issues - Recommendations on media company recruitment materials and media strategy |
| Week 6 | **Media Company Presentation and Discussion:**   - Presentation of revised recruitment materials and discussion   **Discussion Topics**:   - Customer service at clinical trial sites |
| Week 7 | **Presentation Topics:**   - Data Safety Monitoring Boards (DSMB)   **Discussion Topics:**   - Addressing clinical trial study pause - How to communicate to communities about the DSMB and the study pause |
| Week 8 | **Presentation Topics:**   - Media company review of talking points for physicians and outreach   **Discussion Topics:**   - Review of feedback on local COVID-19 recruitment website - Outreach for vaccine trial, and what that looks like post-panel |

Abbreviations: CCP, Community Consultant Panel; NIH, National Institute of Health
